# Evaluation of the sentinel yellow fever surveillance system in Uganda, 2017–2022: strengths and weaknesses

**DOI:** 10.1186/s12879-024-09580-x

**Published:** 2024-07-09

**Authors:** Mercy Wendy Wanyana, Patrick King, Richard Migisha, Benon Kwesiga, Paul Edward Okello, Daniel Kadobera, Lilian Bulage, Joshua Kayiwa, Annet Martha Nankya, Alex Riolexus Ario, Julie R. Harris

**Affiliations:** 1 Uganda Public Health Fellowship Program, Uganda National Institute of Public Health, Kampala, Uganda; 2 Ministry of Health, Uganda National Institute of Public Health, Kampala, Uganda; 3 Arbovirology Department, Uganda Virus Research Institute, Entebbe, Uganda; 4 US Centers for Disease Control and Prevention, Kampala, Uganda

**Keywords:** Yellow fever, Surveillance system, Evaluation, Uganda

## Abstract

**Background:**

Uganda has a sentinel surveillance system in seven high-risk sites to monitor yellow fever (YF) patterns and detect outbreaks. We evaluated the performance of this system from 2017 to 2022.

**Methods:**

We evaluated selected attributes, including timeliness (lags between different critical time points), external completeness (proportion of expected sentinel sites reporting ≥ 1 suspect case in the system annually), and internal completeness (proportion of reports with the minimum required data elements filled), using secondary data in the YF surveillance database from January 2017–July 2022. We conducted key informant interviews with stakeholders at health facility and national level to assess usefulness, flexibility, simplicity, and acceptability of the surveillance system.

**Results:**

In total, 3,073 suspected and 15 confirmed YF cases were reported. The median time lag from sample collection to laboratory shipment was 37 days (IQR:21–54). External completeness was 76%; internal completeness was 65%. Stakeholders felt that the surveillance system was simple and acceptable, but were uncertain about flexibility. Most (71%) YF cases in previous outbreaks were detected through the sentinel surveillance system; data were used to inform interventions such as intensified YF vaccination.

**Conclusion:**

The YF sentinel surveillance system was useful in detecting outbreaks and informing public health action. Delays in case confirmation and incomplete data compromised its overall effectiveness and efficiency.

## Background

Yellow fever (YF) is a vaccine-preventable acute viral haemorrhagic fever, transmitted by *Aedes* mosquitoes from and between non-human and human primates [1]. YF outbreaks can have high case-fatality rates (up to 50%), and there is no specific treatment for infection [2]. Recently, YF has re-emerged in several African countries, including Uganda, that lie in the ‘*yellow fever belt*,*’* characterised by the presence of equatorial rainforest [3–5]. The continued presence of YF is enabled by ongoing low vaccination coverage, inadequate disease surveillance and response, and low commitment to YF prevention initiatives [6].

Since 2016, Uganda reported four YF outbreaks with a total of 68 suspect cases and 21 confirmed cases [7–9]. In these outbreaks, incidence ranged from 3 to 13 cases per 100,000 population with a case-fatality rate of 33% [7, 10]. This re-emergence presents new demands on disease surveillance, especially as Uganda joins the rest of the world to attempt to achieve the World Health Organization’s target to eliminate YF epidemics by 2026 [11].

In Uganda, YF vaccination rates remain low despite the high risk [10], underscoring the need for an effective surveillance system. Surveillance for YF is vital to inform the need for intensified, targeted YF vaccination and to contain outbreaks. Currently, Uganda has both passive and sentinel surveillance systems for YF. The passive surveillance system uses the Integrated Disease Surveillance and Response (IDSR) approach, in which suspected YF cases are routinely reported from all health facilities together with other reportable diseases [12]. In the sentinel surveillance system, seven health facilities in central and western Uganda routinely detect suspected YF cases, collect samples, and send them to the Uganda Virus Research Institute where they are tested for YF [13]. However, the limited access to effective diagnostics for passive surveillance, difficulties in the clinical recognition of the disease, and late reporting [3, 14–16] may limit the overall effectiveness of the YF surveillance systems.

Currently, there is limited evidence on the effectiveness of the YF sentinel surveillance system in Uganda. We described sentinel YF surveillance in Uganda, mapped the surveillance system’s processes, evaluated them to identify strengths and weaknesses, and suggested recommendations aimed at improving YF surveillance in Uganda.

## Methods

### Study setting

The evaluation was conducted in Uganda, a YF-endemic country in Eastern Africa [17]. Uganda is characterised as a high-risk country for YF transmission due to its low vaccination coverage (4% in 2022) and sporadic outbreaks occurring every three to five years [18].

### Study design

We based our evaluation on the US Centers for Disease Control and Prevention’s (US CDC) Updated Guidelines for Evaluating Public Health Surveillance Systems. The guidelines provide a framework with which to assess a system’s performance using key attributes of timeliness, completeness, usefulness, acceptability, flexibility and simplicity [19].

### Study variables and data collection

#### Study variables

For timeliness, we calculated the median time lag between the onset of symptoms and sample collection, the time lag between sample collection and dispatch of the sample to the lab, and the time lag between dispatch to the lab and receipt at the laboratory. For external completeness, we calculated the percentage of the expected YF sentinel sites reporting suspected cases to the YF surveillance system between 2017 and 2022.

Internal completeness was calculated as the proportion of suspected case records with all minimum 10 data elements completed as indicated in the WHO Recommended Surveillance Standards, Second edition [20]. Minimum data elements for case-based reporting for YF include a unique identifier, geographic area (district of residence), date of birth, date of onset of symptoms, ever received YF vaccine, date of sample receipt at the laboratory, tests done, date of result, final classification, and outcome.

We assessed usefulness using stakeholders’ perceptions about actions taken as a result of surveillance outputs. We assessed acceptability using the perceived willingness of key stakeholders to participate in the surveillance system. We assessed flexibility using stakeholders’ perceptions of the ability of the surveillance system to cope with changes. We defined simplicity as perceived ease of performing tasks in the YF surveillance system.

#### Data collection

We obtained data on the YF surveillance system’s purpose, operations, current implementation, and processes using a topic guide and process map. Additionally, we reviewed relevant documents, including guidelines and reports, to obtain more information on the system’s processes and current implementation using a document review guide. We then assessed the surveillance system’s timeliness, external completeness and internal completeness using data from the YF surveillance database. Furthermore, we assessed the system’s usefulness, acceptability, flexibility, and simplicity. To assess these attributes (usefulness, acceptability, flexibility, and simplicity) we purposively selected key stakeholders at the Uganda Virus Research Institute, the Ministry of Health and sentinel site health facilities and conducted key informant interviews.

### Data management and analysis

We used Epi info 7 software (CDC, Atlanta, USA) to analyse quantitative data using descriptive statistics. We assessed the trend in the time lags between onset of symptoms and sample collection and the time lag between collection of samples, and dispatch to the laboratory using the Mann-Kendall test. We used the chi-square test to assess the difference in positivity rates across regions.

For qualitative data, we transcribed audio-recorded interviews and analysed data using a deductive thematic analysis in Atlas ti 7 software (Scientific Software Development GmbH, Berlin Germany). Transcripts were coded and themes were generated based on pre-conceived themes on the YF surveillance system’s usefulness, acceptability, flexibility, and simplicity.

## Results

### Description and operations of the YF sentinel surveillance system, Uganda, 2017–2022

YF sentinel surveillance is part of the arbovirus surveillance system established by the Department of Arbovirology, Emerging and Re-Emerging Infectious Diseases at UVRI in 2013. The surveillance system aims to (1) prevent outbreaks of arboviral diseases through early detection, diagnosis, and identification within the region; (2) provide risk assessments of the different emerging viruses (transmission, spread, human impact); and (3) recommend and implement public health measures for control where possible.

**Fig. 1:**
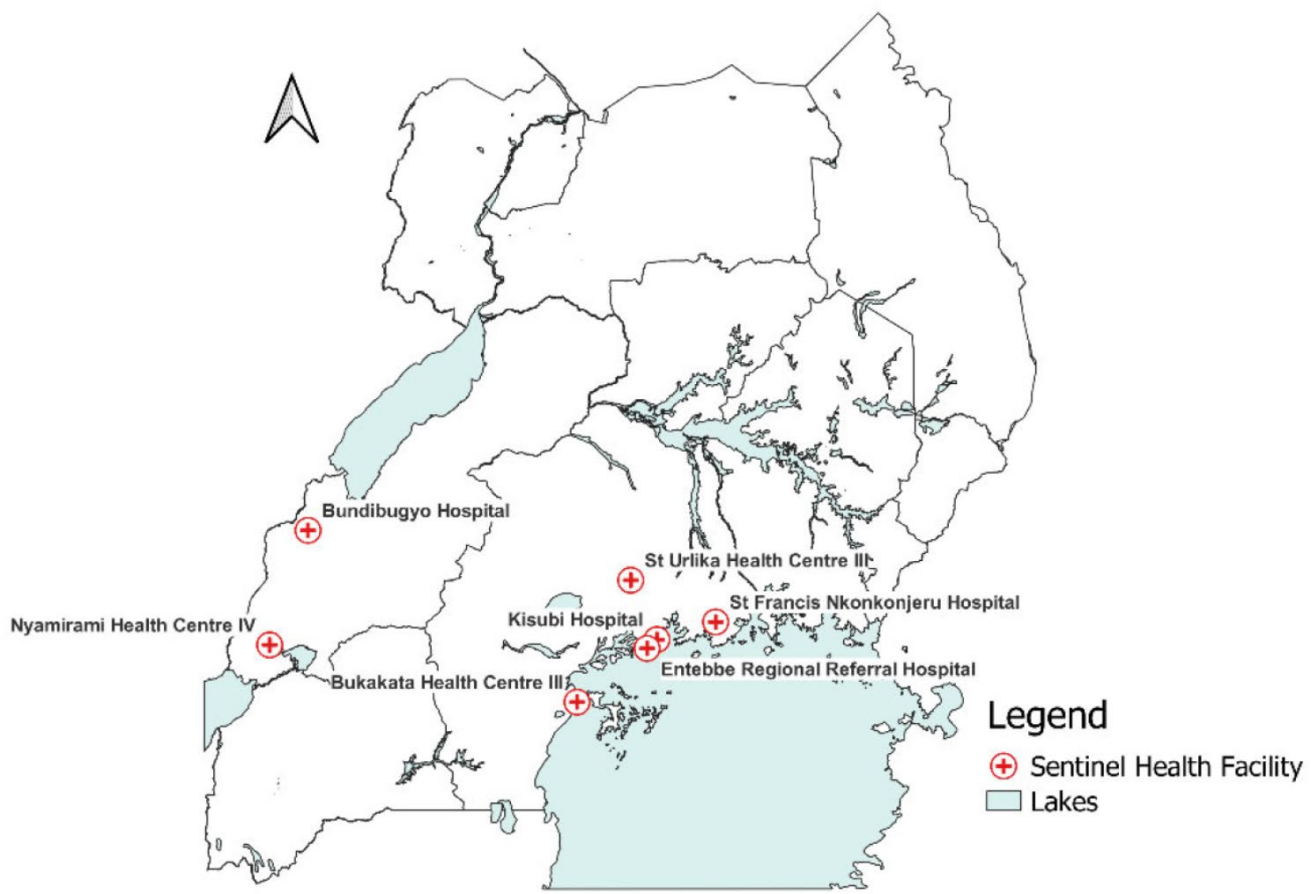
Location of YF sentinel surveillance sites in Uganda, 2017– 2022

For YF, the surveillance system has seven sentinel sites (Fig. 1). These sites were enrolled in a phased manner, starting with St. Francis Nkonkonjeru in 2013; Bukakata Health Centre III, Kisubi Hospital, St Ulrika Health Centre III, Entebbe Regional Referral Hospital, and Nyamirami Health Centre in 2017; and Bundibugyo Hospital in 2020. These sites were selected based on previous entomological studies that identified the presence of YF vectors (*Aedes aegypti* mosquito species) carrying viruses of the *Flaviviridae* family in the areas where these facilities are located. The surveillance systems target at-risk YF populations based on their proximity to these “high-risk environments.”

#### Processes in the YF sentinel surveillance system, Uganda, 2017–2022

The YF sentinel surveillance system starts with a clinician in a sentinel site suspecting YF based on a case definition of acute onset of fever that is not responsive to malaria treatment. However, clinicians reported often using their clinical judgement to identify suspect YF cases instead of the case definition. A blood sample of ≥ 2 ml is collected from the patient in a sterile vacutainer and blood is centrifuged to separate serum. The serum is separated and stored in a liquid nitrogen tank until the collection date (once every month). Samples are transferred to UVRI for testing by the UVRI YF sentinel surveillance project team. Additionally, every suspected viral haemorrhagic fever sample submitted to UVRI that is negative for other viral haemorrhagic fevers is submitted for YF testing. Samples are tested using the YF testing algorithm in the WHO guidelines [21]. Interpretation of results is based on vaccination history, date of onset of symptoms, date of sample collection, and travel history. A positive PCR test from a person with no history of recent YF vaccination OR positive anti-viral immunoglobulin M (IgM) test in a person with no history of vaccination and Plaque Reduction Neutralisation Test (PRNT) titre with YF antibodies detected at ≥ 4 times greater than PRNT titres for dengue, West Nile and Zika flaviviruses is considered a positive test for YF. The laboratory testing process takes a total of 2 weeks. Results are entered into the database and shared with MoH and WHO every week.

#### Personnel and tasks in the YF sentinel surveillance system, Uganda, 2017–2022

There were various personnel responsible for the different tasks within the sentinel surveillance system (Table 1). These include clinicians, laboratory technicians, drivers, laboratory staff, project management staff, and information analysts.

**Table 1:** Personnel and tasks in the YF sentinel surveillance system, Uganda, 2017–2022

Tasks	Personnel	Level
Case detection	Clinicians	Sentinel Site
Collection, initial preparation of samples, and storage of samples	Laboratory technicians	
Transportation of samples	Drivers	UVRI
Testing of samples	Laboratory staff	
Train sentinel surveillance staff to accurately collect, store, and process samples	Project management staff	
Track information in database to identify positive test results	Information analysts	Ministry of Health Public Health Emergency Operations Centre
Routine analysis of surveillance data over time	No personnel assigned for this task	

### Availability of surveillance guidelines, documents, and protocols for the YF surveillance system, Uganda, 2017–2022

All YF sentinel sites reported having received a written protocol and guidelines on sample collection and storage at the establishment of the sentinel sites. However, at the time of the visit, only 3 of the sites had a copy of these documents available.

### Data flow, reporting, feedback mechanism, and data management for the YF surveillance system, Uganda, 2017–2022

**Fig. 2:**
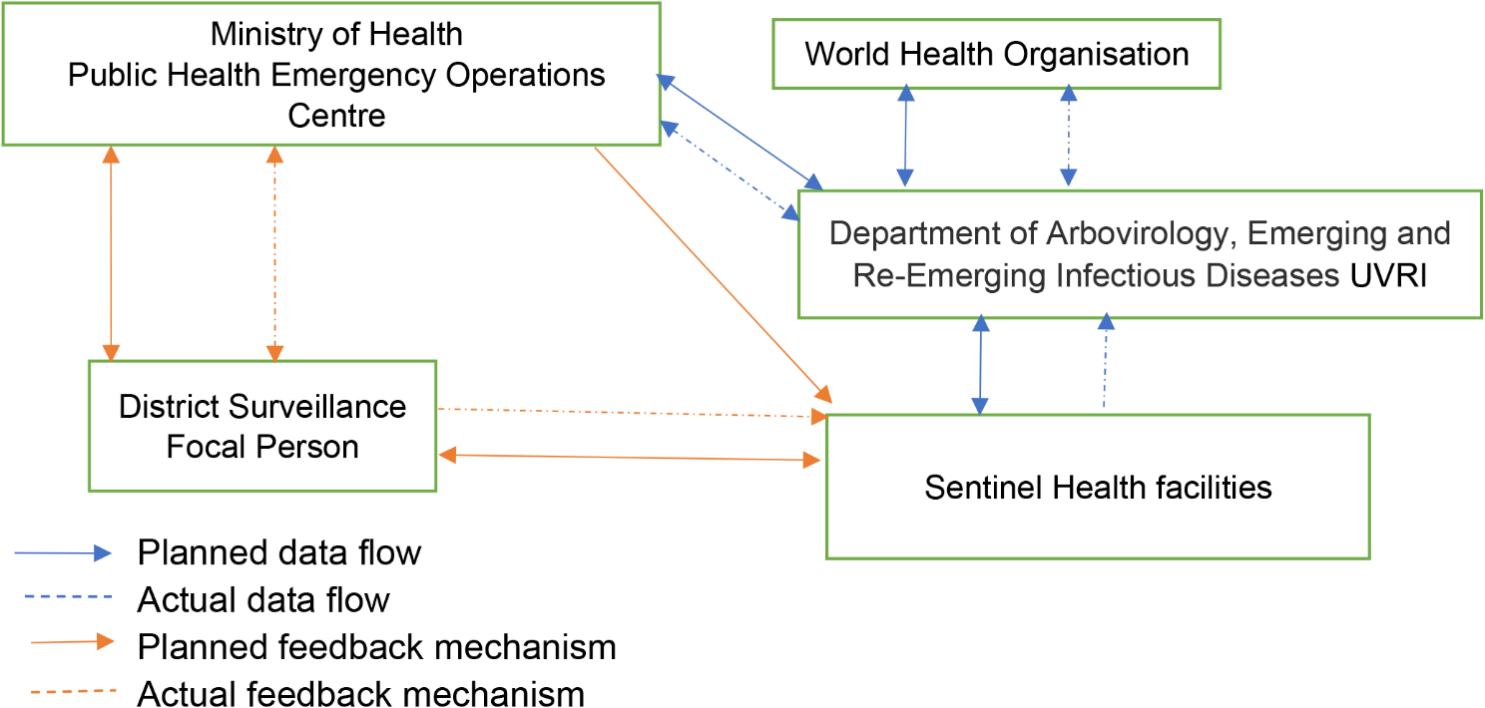
Data flow reporting and feedback mechanism in the YF surveillance system, Uganda, 2017–2022

Sentinel health facilities collect data on suspected cases using the paper-based UVRI Viral Haemorrhagic Fever case investigation form (Fig. 2). Additionally, a summary of the patient information (name, residence, age, and sample collection date) is recorded in a book at the health facility. All completed, hard copy case investigation forms are submitted to UVRI, together with their corresponding sample. Following testing, data are entered into an EpiInfo database and sent in a Microsoft Access data to the Ministry of Health Public Health Emergency Operations Centre (MoH PHEOC) and the World Health Organization every week. The MoH PHEOC reviews the database every week, if there are any positive cases reported, the MoH PHEOC notifies the District Surveillance Focal Persons through email, phone calls, and SMS Alerts. Data are not routinely analysed by person, place, and time.

### Performance of the sentinel YF surveillance system based on key attributes, Uganda, 2017–2022

Over the review period, a total of 3,073 suspect cases with samples collected were reported in the YF sentinel surveillance system. Of these, 15 (0.49%) were confirmed as YF cases.

### Timeliness

**Fig. 3:**
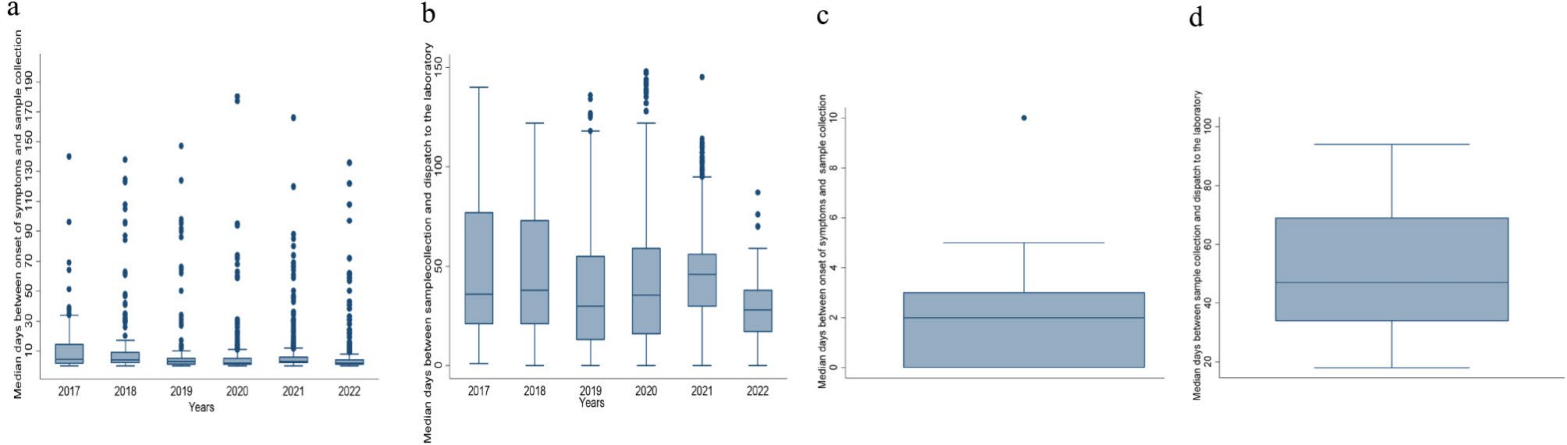
Time lags between onset of symptoms, sample collection and dispatch of samples to the laboratory, **A** Overall time lag between the onset of symptoms and sample collection, 2017–2022, **B** Overall time lag between sample collection and collection of sample and dispatch to the laboratory, 2017–2022, **C** time lag between the onset of symptoms and sample collection, for confirmed cases 2017–2022, **D** The time lag between sample collection and collection of sample and dispatch to the laboratory for confirmed cases, 2017–2022

The overall median number of days between the onset of symptoms and sample collection was 3 [Interquartile Range (IQR = 1–5)]. Confirmed cases had a shorter median time between onset of symptoms and sample collection was 2 [Interquartile Range (IQR = 1–10)] (Fig. 3C). The overall median number of days between sample collection and dispatch of the sample to the lab was 37 (IQR = 21–54). Confirmed cases had a longer median time between sample collection and dispatch of sample to the laboratory was 47 [Interquartile Range (IQR = 34–69)] (Fig. 3D). The shortest time lag occurred between sample dispatch and receipt at the laboratory (median: 1 day, IQR = 0–1). From 2017 to 2022, the overall median time lag between onset of symptoms and sample collection reduced from 5 to 2 days (*p* = 0.001) (Fig. 3A). Similarly, the overall time lag between collection of samples and dispatch to the laboratory reduced from 36 to 28 days (*p* = 0.002) (Fig. 3B).

### Completeness

#### External completeness

With the exception of 2022, there was an increase in the number of samples submitted each year (Table 2). From 2017 to 2019, external completeness was 100%, with all the YF sentinel sites reporting at least one suspected case into the system each year. From 2020 to 2022, one site did not report any suspected cases. During this period, external completeness was 86%. The central region had higher overall positivity rates in comparison with the western region, 0.75% Vs 0.12%,*p* = 0.014.

**Table 2:** External completeness of the YF sentinel surveillance system, Uganda, 2017–2022

Region	Sentinel Site							
		2017	2018	2019	2020	2021	2022	Total
Central	*St Francis Nkonkonjeru*							
	Total number of samples	67	84	69	68	213	100	601
	Positive samples	0	0	0	2	0	0	2
	Percent positive(%)	0	0	0	2.9	0	0	0.33
	*Bukakata HC III*							
	Total number of samples	30	70	51	58	99	54	362
	Positive samples	0	0	1	1	0	1	3
	Percent positive(%)	0	0	2	1.7	0	1.9	0.83
	*St Urlika HC III*							
	Total number of samples	9	40	68	70	98	87	372
	Positive samples	0	0	2	0	1	1	4
	Percent positive(%)	0	0	2.9	0	1.02	1.1	1.1
	*Kisubi Hospital*							
	Total number of samples	15	49	84	103	135	104	406
	Positive samples	0	0	0	1	2	2	5
	Percent positive(%)	0	0	0	0.97	1.5	1.9	1.2
	*Entebbe RRH*							
	Total number of samples	10	30	70	0	0	0	0
	Positive samples	0	0	0	0	0	0	0
	Percent positive(%)	0	0	0	0	0	0	0
Western	*Nyamirami HCIV*							
	Total number of samples	2	75	110	168	277	113	745
	Positive samples	0	0	0	2	0	0	2
	Percent positive(%)	0	0	0	0.6	0	0	0.13
	*Bundibugyo Hospital*							
	Total number of samples				106	163	124	393
	Positive samples				2	0	0	0
	Percent Positive				0.6	0	0	0

HC- Health Centre, RRH-Regional Referral Hospital

^*^Bundibugyo Hospital sentinel site was added in 2020,therefore has no data for 2017–2018

### Internal completeness

**Fig. 4:**
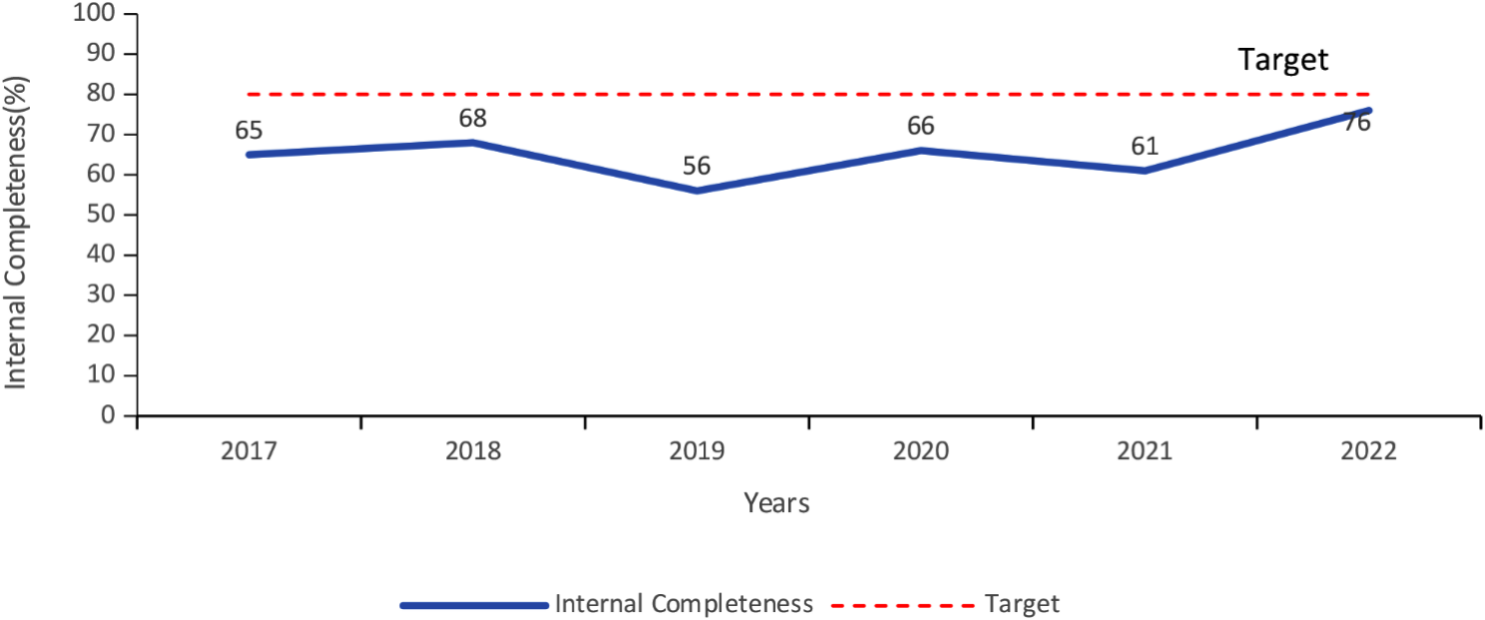
Internal completeness of data reported in the YF sentinel surveillance system, Uganda, 2017–2022

Overall internal completeness was 65%, ranging from 56% in 2017 to 76% in 2022 (Fig. 4). Of the ten data fields analysed for internal completeness, unique identification number, age, YF IgM results, and PRNT tests were the most often completed data fields (> 95%). Vaccination status, IgM result release date, and PRNT result release dates were the least reported fields at < 1%.

### Usefulness

According to the respondents, the YF sentinel surveillance system met its objectives of detecting YF outbreaks and on-going YF transmission in communities and informing prevention and control interventions. Of the 21 confirmed cases detected during 2019–2022, 15 (71%) were detected through the YF surveillance system.

*“…the sentinel YF surveillance system is very helpful in detecting YF cases…in previous outbreaks*, *most of the index YF cases were identified through the sentinel surveillance system….”*

#### MoH official, Public Health Emergency Operations Centre

Additionally, key informants reported that the sentinel YF surveillance system provided data that helped guide decisions on the selection of interventions for YF prevention and control.

…*the YF sentinel surveillance system has enabled us to identify which communities are at risk of YF transmission… as a result*, *these have been prioritised in the intensified YF vaccination campaigns last year ….*

#### MoH official, Department of Integrated Epidemiology, Surveillance and Public Health Emergencies

…currently we use data obtained from the YF sentinel surveillance system to advocate for the introduction of the YF vaccine into the routine vaccination schedule…this will start soon …and if we didn’t have those data to justify our recommendations, they would not be taken up….

#### MoH official, Department of Integrated Epidemiology, Surveillance and Public Health Emergencies

##### Acceptability

Respondents reported willingness, commitment, and interest in participating in the YF surveillance system. In particular, stakeholders at YF sentinel sites reported that their active involvement in the running of the YF surveillance system made it more acceptable to them.

…*We are very committed and willing to participate in the YF surveillance system. For no extra pay*,* we continuously assess and report any suspected YF cases to UVRI….*

#### Health worker at a YF sentinel site

… all of us, including community members, are actively involved in the YF surveillance system and our contributions are valued…we have regular discussions with project managers from UVRI on how we can improve YF surveillance….

#### Health worker at a YF sentinel site

##### Flexibility

Respondents reported uncertainty about the YF sentinel surveillance system’s capacity to adapt to the changing surveillance needs. Reasons cited for this uncertainty included not having experienced a change in YF surveillance needs, and lack of funding to support changes even if needed.

…*We have not experienced any changes in the YF surveillance system…we*,* therefore*,* cannot tell how flexible the system is…*.

#### MoH official, department of integrated epidemiology, surveillance and public health emergencies

“…*Due to limited funding*,* the sentinel YF surveillance system cannot be as flexible as we would love it to be…. for example*,* we cannot have more sentinel sites even if we wanted. For example*,* we experienced a YF outbreak in the northern region of the country in 2016 suggesting a need for enhanced surveillance in this area but no surveillance sites have been set up in this area due to [lack of] funding…”*.

#### MoH official, Department of integrated Epidemiology, Surveillance and Public Health Emergencies

##### Simplicity

The respondents’ tasks within the sentinel surveillance system were reported as being easy to do.

…*the processes are easy and simple…all you have to do is the case investigation form which is easy to fill and wait for UVRI to pick the form*….

#### Health worker at a YF sentinel site

## Discussion

Prevention and control of YF outbreaks require a reliable and effective surveillance system. We described Uganda’s YF sentinel surveillance system, mapped the surveillance system’s processes, and evaluated them to identify strengths and weaknesses. During 2017–2022 in Uganda, most sentinel surveillance sites actively submitted samples, with an increase in samples submitted over time. Most sites submitted at least one positive sample during the evaluation period. We noted limited use of case definitions, with clinicians more often using individual clinical judgement and expertise to identify suspected cases, as well as delays in case confirmation and incomplete data.

In malaria-endemic areas like Uganda where acute febrile illnesses are common, non-malarial acute febrile illnesses such as YF may be missed. In these settings, laboratory-based arbovirus sentinel surveillance improves detection of YF which is key for achieving the WHO target of eliminating YF epidemics by 2026 [22]. Most sites submitted samples over the evaluation period. Continuous submission of samples by sentinel sites is critical for the functionality of YF surveillance system [23]. One site, Entebbe Regional Referral Hospital, stopped submitting samples in 2020, during a period when it was restricted to providing only COVID-19 management services. We observed an increase in the number of samples over time, with positivity rates similar to those previously documented in Uganda and the sentinel YF surveillance system in the United Republic of Tanzania [24, 25]. Generally, sites in the central region had higher positivity rates compared to sites in the western region. This supports previous findings that have indicated a higher seroprevalence, and likely more circulation, of YF in the central region [25].

Interestingly, some sites had no cases identified throughout the period despite submission of large numbers of samples. It is possible that the limited use of case definitions and reliance on clinical judgement could have contributed to this, especially with less skilled health workers [26]. In Uganda, lower cadres sometimes perform duties of specialised health workers with minimum supervision [27, 28]. In this situation, ‘over-testing’ as a result of using clinical judgment instead of the case definition could waste resources in an already resource-constrained system [29]. This may also be true even at the sites submitting some positive samples. However, the sites without any positives also generally submitted fewer samples than the sites with positive samples. The use of simplified validated standard case definitions provided in the Uganda National Technical Guidelines on Integrated Disease Surveillance and Response 3rd Edition could standardise diagnosis and improve identification of suspected cases within the surveillance system [12]. This will improve the performance of the surveillance system as correct knowledge and use of standard case definitions among health workers at sentinel sites is essential for an effective surveillance system. Further investigations into the reasons for the lack of positive samples at these sites could shed additional light on these findings.

Although most sites submitted samples, internal completeness of data was very low and below the targets for an effective surveillance system (100% completeness for the minimum data elements of unique identifier, district of residence, date of birth, date of onset of symptoms, ever received YF vaccine, date of sample receipt at the laboratory, tests done, date of result, final classification, and outcome) [20]. In particular, vaccination history was almost never filled out. This variable is critical to interpretation of YF testing results [30]; because receipt of the YF vaccine can cause a positive antibody result even in an uninfected person, positive test results among vaccinated persons tend to be discounted [21, 31]. A lack of information on YF vaccination status can incur investigation costs and use resources unnecessarily. Beyond this, incomplete data can make follow-up investigations challenging if a patient cannot be located. Failure to value data quality and lack of feedback and supervision among health workers may contribute to incomplete data even when forms are reported as easy to complete [32, 33]. Incomplete data gaps may remain unaddressed where there is limited feedback and data flow to sentinel sites and districts as identified in this assessment. We recommend regular data quality audits, mentorship and supportive supervision to improve data completeness data within the surveillance system [34].

We found sizeable time lag between sample collection and shipment to the laboratory which was higher than the expected WHO threshold [30, 35]. Because of reported resource constraints, samples took at least 3 weeks to be shipped to the laboratory instead of the recommended < 24-hour timeframe. Similar findings have been reported in the Central African Republic, and other African YF endemic countries with challenges in transportation of laboratory samples [36]. Such delays may hinder the timely detection of YF outbreaks. Due to this long duration of confirming a suspected case, YF cases would have either healed or died by the time a case is confirmed, limiting the usefulness of the YF surveillance system. To address such delays the YF surveillance system could adopt the Uganda laboratory hub system, which was used to reduce turnaround times for HIV test results and transport costs by half improving early infant diagnosis [37]. Preliminary findings from a project improving yellow fever testing turn-around time at a Health Centre III indicated that using the public Uganda laboratory hub transport system reduced time between sample collection and shipment to the laboratory to < 7 days between May to June 2023.

While stakeholders reported that the system was useful in identifying individual cases for response, sentinel surveillance systems often are designed to monitor patterns. Despite this, data generated by the sentinel YF surveillance system were not routinely analysed. This was attributed to the lack of personnel assigned to data analysis roles. The lack of routine analysis of YF sentinel surveillance data limits its usefulness in monitoring disease trends and using these data for decision-making [35]. Specifically, this may result in missed opportunities in tracking patterns of YF transmission over time. Additionally, analysis of YF sentinel surveillance data is vital to achieving WHO’s goal to eliminate yellow fever epidemics by 2026 [38]. Enhanced efforts to carry out periodic analysis of the sentinel data could improve the usefulness of the system.

### Limitations

Although the qualitative approach to evaluating system attributes elicited respondents’ in-depth perspectives, these could have been biased due to social desirability. The positive predictive value of the system could not be ascertained as only the gold standard measurement of YF diagnostics was used.

## Conclusion

In conclusion, the yellow fever sentinel surveillance system had sentinel sites in areas with documented YF circulation in Uganda that facilitated detection of most YF outbreaks in Uganda. However, gaps in delays in case confirmation, incomplete data and inconsistent use of case definitions reduced its overall effectiveness and efficiency.

## Data Availability

The datasets upon which our findings are based belong to the Uganda Public Health Fellowship Program. For confidentiality reasons, the datasets are not publicly available. However, the datasets can be availed upon reasonable request from the corresponding author with permission from the Uganda Public Health Fellowship Program.

## Abbreviations

ELISA: Enzyme-Linked Immunosorbent Assay
IDSR: Integrated Disease Surveillance and Response
MoH: Ministry of Health
PHEOC: Public Health Emergency Operations Centre
PRNT: Plaque Reduction Neutralisation Test
UVRI: Uganda Virus Research Institute
WHO: World Health Organisation
